# *Cryptosporidium* in human-animal-environment interphase at Adama and Asella areas of Oromia regional state, Ethiopia

**DOI:** 10.1186/s12917-022-03497-w

**Published:** 2022-11-14

**Authors:** Kassahun Berhanu, Dinka Ayana, Bekele Megersa, Hagos Ashenafi, Hika Waktole

**Affiliations:** 1 College of Veterinary Medicine, Department of Parasitology and Pathology, Jigjiga University, P.O. Box 1020, Jigjiga, Ethiopia; 2 College of Veterinary Medicine and Agriculture, Department of Pathology and Parasitology, Addis Ababa University, P.O. Box 34, Bishoftu, Ethiopia; 3 College of Veterinary Medicine and Agriculture, Department of Microbiology, Immunology and Veterinary Public Health, Addis Ababa University, P. O. Box 34, Bishoftu, Ethiopia; 4 Addis Ababa University, Akililu Lema Institute of Pathobiology, Addis Ababa, Ethiopia

**Keywords:** Dairy cattle, *Cryptosporidium*, Human, Manure, Occurrence, Sheep, Ethiopia

## Abstract

**Background:**

In Ethiopia, several studies have reported the occurrence of *Cryptosporidium* infection in young calves, lambs, and HIV positive patients. However, research on cattle and sheep of all ages, as well as humans, has been limited.

**Methods:**

A cross-sectional study was conducted to investigate the occurrence of *Cryptosporidium* in dairy cattle, sheep, humans, and manure samples. Dairy cattle and sheep were stratified according to their age groups and randomly sampled. Stool samples from volunteered dairy farm workers and rural household members were collected purposefully. A semi-structured questionnaire was also used to collect information about manure handling practices and socio-demographic variables. All collected samples were processed with the sugar floatation technique and the modified Ziehl–Neelsen staining method. Logistic regression was used to analyze the association of risk factors with the occurrence of *Cryptosporidium in study subjects* (*p* < 0.05).

**Results:**

The overall prevalence of *Cryptosporidium* in dairy cattle, sheep, humans, and manure samples was 20.5%, 14%, 16%, and 13.20%, respectively. *Cryptosporidium* infection was significantly higher in dairy cattle aged 1–12 months and 13–36 months with odds of 3.48 and 3.07 times higher, respectively, compared to others. Similarly, its occurrence was 2.69 times higher in sheep aged 1–6 months than those above 6 months. And also, a higher average oocyst count (above 10 oocysts) per-field was observed in cattle aged 1–12 months, followed by sheep aged 1–6 months. Furthermore, the likelihood of infection was 13 times greater in farm workers compared to household members of smallholder farmers. In addition, the occurrence of oocysts was 22.8 times higher in manures from dairy cattle than that of sheep. About 16.8% of the study respondents had manure disposal pit, 98.1% of them used manure as fertilizer for crop and vegetable production without any treatment.

**Conclusions:**

The study revealed the occurrence of *Cryptosporidium* infection in all age groups of dairy cattle and sheep, humans engaged in animal production. Occurrence of *Cryptosporidium* in manure suggests it potential contamination of environment and water sources.

## Background

*Cryptosporidium* is a unicellular protozoan parasite that causes cryptosporidiosis in a variety of animals, including humans. Though the parasite was discovered by Tyzzer [1] in laboratory mice in 1907, it’s the clinical significance in animals and humans was not recognized for 70 years, until the parasites were discovered in an 8-month-old calf with chronic diarrhea in 1971 [2]. Later, in Australia, the infection was discovered in lambs with diarrhea [3], and further investigations on natural and experimental infections have established its role as a key etiological agent in bovine diarrhea [4].

According to a recent study, more than 44 *Cryptosporidium* species have been identified [5]. From these, *Cryptosporidium parvum*, *Cryptosporidium bovis*, *Cryptosporidium andersoni*, and *Cryptosporidium ryanae* are the major species infecting cattle. Sheep are infected with three main *Cryptosporidium* species; *Cryptosporidium parvum, Cryptosporidium ubiquitum*, and *Cryptosporidium xiaoi*, and humans are mostly infected with *Cryptosporidium parvum* (the most zoonotic species), and *Cryptosporidium hominis* [6]. Other *Cryptosporidium* species are also reported from different animals, and each of which has its own prevalence, geographic range, and public health significance [7].

The infection is transmitted to animals and humans orally through the ingestion of sporulated oocysts. The sexual and asexual life cycles are completed in the same host (monoxenoes) and have a unique location within the host cell, situated between the cytoplasm and the cell membrane.

[8], [9]. Even though *Cryptosporidium* infection causes clinical disease in neonatal calves and lambs, there is evidence of reduced milk production in cows during shedding of *Cryptosporidium andersoni* [10]. The economic losses associated with this disease are not only due to mortality but also to production loss, retarded growth, and the cost of treatment [11].

In particular, neonatal calves and lambs are vulnerable to *Cryptosporidium* infection and shed millions of oocysts, resulting in enormous environmental contamination and a risk of infection to other animals and humans [12]. Meanwhile, asymptomatic weaned and adult cattle also excrete oocysts into the environment [13]. According to Scott et al. [14], a single adult bovine might possibly excrete more than 36 million oocysts every day. Other researchers indicated that the sub-clinically infected ewes are also a source of infection for lambs, especially during the peri-parturient period [15]. Without adequate control, this contamination represents a human health hazard because infected animals could shed up to 10^7^ oocysts per gram of feces [16].

Oocysts are resistant to environmental conditions and survive for a months in environments and animal manures under cool and wet conditions. Infected animal manure also serves as a significant reservoir for *Cryptosporidium* infection [17]. Other studies indicated that contaminated manures from dairy or beef operations are substantial sources of *Cryptosporidium* oocysts for humans and animals, unless manure management or treatment measures are implemented to decrease oocyst viability or transmission to water [18]. Surface transfer from land-applied manures or leaching through the soil to groundwater are two additional mechanisms of transfer of the pathogen to drinking or recreational water, in addition to direct fecal deposition. Runoff from polluted field might act as a vehicle for *Cryptosporidium* oocysts to enter water sources. As a result, cattle farms might be a major source of *Cryptosporidium* infection for humans and other animals [19].

Investigation of *Cryptosporidium* infection in apparently healthy adult ruminants is vitally important as they act as reservoirs and become sources of infection for young animals [20]. In addition, infected animal manure serves as a reservoir and favourable for a long period survival of *Cryptosporidium* oocyst in the environment. When pathogen-laden manure is sprayed as fertilizer on grazing fields, there is a risk of environmental contamination and increase animal exposure. Untreated manure use as fertilizr for vegetable cultivation also poses a public health threat. Additionally, Intense rainfall results in surface water runoff, which can transport oocysts from farms into surrounding watersheds (water sources), resulting in high pathogen loads and more potential for human infection [21].

The prevalence of *Cryptosporidium* infection ranges from 10.8 to 27.8% [22], [23] and 2.1 to 22.2%[22], [24] in calves and lambs, respectively. On the other hand, Hailu et al. [25] reported the highest prevalence (46%) of *Cryptosporidium* infection in humans having contact with animals. The majority of the reports in Ethiopia were from young calves, lambs, and HIV positive patients, and with limited information for adult animals [23, 26, 27]. Previous studies have mostly focused on young animals and humans in central parts of Ethiopia, whereas its infection in adult cattle and sheep has not been comprehensively investigated. In addition, the occurrence of infection in animal manure has not yet been documented in study areas. Therefore, this study was to examine the occurence of *Cryptosporidium* infection in all ages of cattle, sheep, and humans as well as the presence of oocysts in animal manures. Additionally, the potential risk factors and the intensity of *Cryptosporidium* oocysts were also determined.

## Materials and methods

### Study areas

**Fig. 1:**
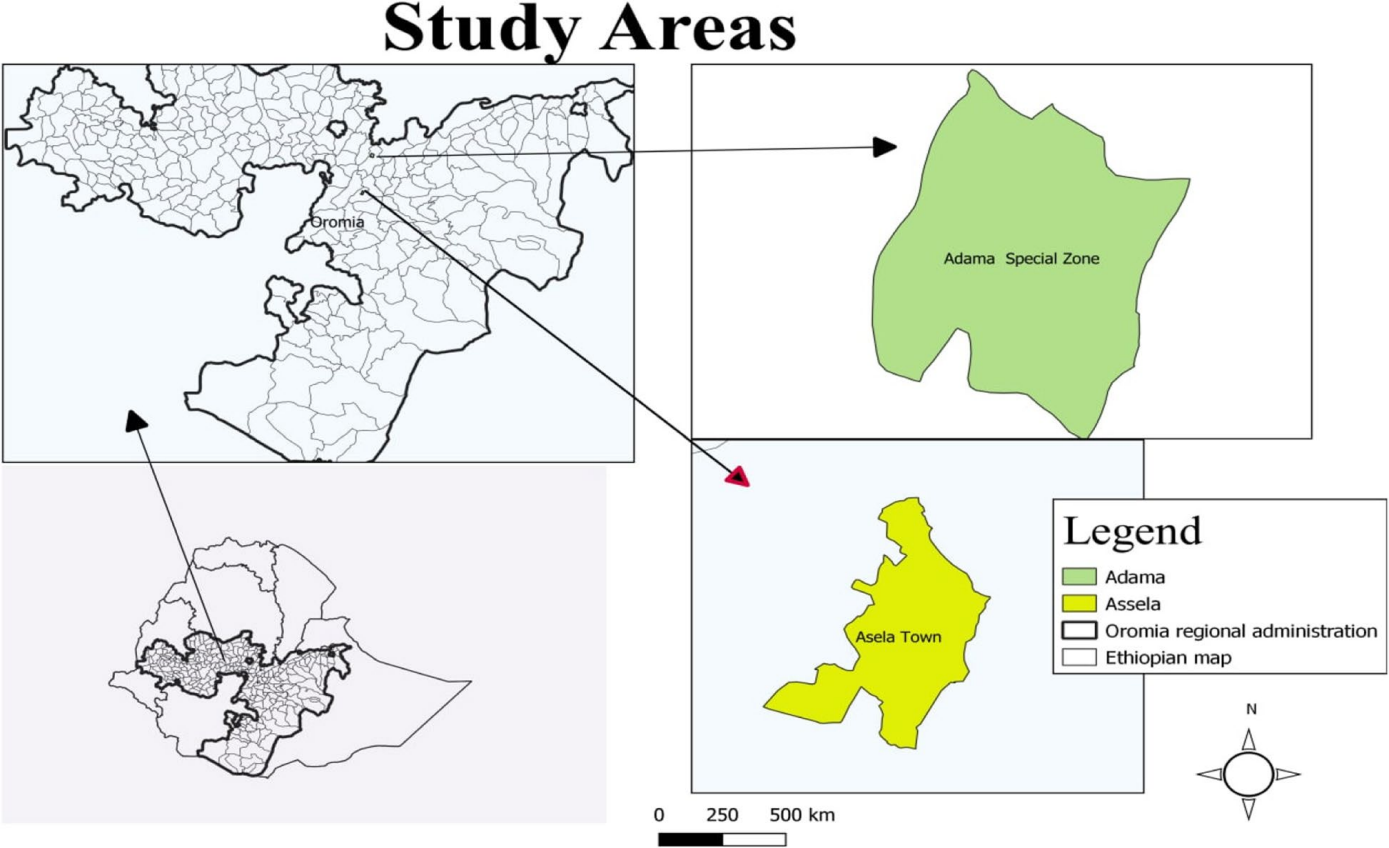
Map of Ethiopia showing the location of study areas of Adama and asella Towns. The green and orange colors indicates Adama and Asella towns respectively

The study was conducted in two agro ecologies: Adama representing midland and Asella (highland) areas of Oromia Regional State (Fig. 1). Adama Town located 99 km south-east of Addis Ababa, in the rift valley, found between 8° 33' and 8° 36'N latitude and 39° 11′ 57" to 39° 21′ 15"E longitude. The town has an average altitude of 1712 m above sea level, and receives an annual range of rainfall ranging 600 to 1150 mm, and temperature range between 12 and 33 °C. The livestock populations of the town and suburb area are estimated to be 103,440 cattle, 45,554 sheep, 54,112 goats, and 87,341 poultry [28].

Asella town located 175 km to the south-east of Addis Ababa in Arsi zone. The town has altitude range of 1780 and 3100 m above sea level, lies between longitude and latitude of 6° 59 to 8° 49 N and 38° 41 to 40° 44 E, respectively. The average annual rainfall in the area is 1200 mm, with minimum and maximum temperature of 5 °C and 28 °C, respectively. The population of livestock Asella and its surrounding area was estimated at 82,190 cattle, 52,292 sheep, 11,479 goats, and 162,015 poultry [29]. Even though the study areas are known for their livestock population and products (milk, meat, skins, and hides), the occurrence of cryptosporidiosis in animals, humans, and the environment has not been fully investigated.

### Study population

The study population comprises cattle and sheep selected from dairy farms and smallholder farmers. Animals from all age groups and sexes were chosen for sampling. The dairy cattle on the selected farm are managed under intensive to semi-intensive production systems. The Holstein breed is the most common among farmers, followed by crosses and the Borana cattle. Local sheep breeds reared under an extensive production system were also included for sampling. Furthermore, people working on dairy farms and household members of smallholder farmers having close contact with animals were targeted to examine the presence of *Cryptosporidium* infection in humans.

The body condition of dairy cattle and sheep were categorized according to Kilopic et al. [30] and Thomson and Mayer [31], respectively. The age of dairy cattle was estimated by looking at their ear tags; while that of sheep was conventionally recorded by asking their owners. Criteria were developed to evaluate and categorize the hygiene of dairy farms as good (separate calf pens, dry, spacious, no feces, cleaned daily, calves clean, clean floor) and poor (separate calf pens, but wet, washed occasionally, feces present on the floor and on calves).

### Study design and sample size

A cross-sectional study design was conducted from October 2021 to April 2022 to investigate the occurrence of *Cryptosporidium* infection and associated risk factors in dairy cattle, sheep, humans, and manure in the study area. The required sample size for the study was determined using the formula given by Thrusfield [32]. From previous data, the prevalence of *Cryptosporidium* infection in dairy calves and lambs was 18.6% [33] and 15.4% [34], respectively. Using the previous reports as an expected prevalence, 95% confidence interval, and 5% absolute precision, 234 dairy cattle and 200 sheep were recruited for this study. Additionally human stool from voluntary individuals and manure samples (from environment) were sampled from the dairy farms and smallholder households. Hence, pooled manure samples (n = 68) were sampled from half of the selected dairy farms and smallholder households who have piled manure in their compounds.

### Sampling method

Convenience sampling methods were employed to select kebeles and dairy farms in collaboration with Adama and Asella Town agricultural bureaus and farm owners. Location of study farm and the total number of animals found per farms farms were obtained from the town agricultural bureau. Accordingly, a total of six kebeles namely Burqa Cilalo, Ankaka Qonnicha and Gonde from Asella, Soolee, Dhawata, and Wonji from Adama were selected, representing a total of 22 kebeles found in both study areas. After the selection of Kebeles and dairy farms, animals were randomly picked from selected farms considering each age group in the sampling. An attempt was made to proportionally sample the animals from the dairy farms depending on herd size. Age of animals was categorized into three groups: 1–12 months, 13–36 months, and above 36 months from which Comparable number of animals were sampled from each of the age category. On average, 40 animals per farm and 13 animals per age group were sampled to meet the required sample size. Similar sampling method (convenience) was also applied for sheep sampling in addition to using participatory approach for the selection of household owned sheep. A total of six kebeles were selected from which 100 smallholder farmers were sampled by considering their sheep ownership and willingness to participate in the study. We applied a stratified sampling to select animals from the two age groups: 1–6 months, and above 6 months. Then, two sheep per household, and one animal from each age group were randomly sampled.

A purposive sampling method was applied for sampling peoples working in dairy farms and suburb small holder farming household members for stool sampling. Before sampling, the people were informed about the objective of the study and asked for their consent. Though an attempt was made to collect stool at least from one individual per dairy farm and from 100 smallholder farmers, about 56 stool samples were actually collected from consented people who were willing to cooperate to give stool samples.

During sampling, important information about the age of animals, body condition, sex, species, breed, agroecology, fecal consistency, and their management systems were recorded from face-to-face interviews with farm owners and animal attendants. In addition, history of contact with animal feces and manure, place of manure disposal, use of manure, frequency of manure disposal, and age of people participating in the study were also recorded on data collection sheet.

### Sample collection

About 10 g of fresh fecal samples were collected from the rectum of dairy cattle and sheep using disposable gloves. Similarly, 10 g of fresh stool samples were taken from voluntary people by human health laboratory technicians working in the area. In addition, 20 g samples of manure were collected from thoroughly mixed manure storages. The fecal, stool and manure samples were placed in a sterile, airtight plastic bottle. After labelling, the samples were transported in an ice box to the parasitology laboratory of Addis Ababa University, college of veterinary medicine and agriculture. The samples that were not examined on the same day were stored in the refrigerator at + 4 ^0^C.

### Laboratory examination

The samples were processed using Sheather sugar solution and modified Ziehl–Neelsen staining methods following the laboratory safety rules to minimize the risk to infection (viable oocyst). Briefly, samples were examined by Sheather’s sugar solution of 1.27 specific gravity to detect *Cryptosporidium* oocyst microscopically as described by Trotz-Williams et al. [35]. About 3 g of feces were diluted in 42 ml of sugar solution and passed through sieve gauze to remove the solid particles. The concentrated solution was poured into a 15 ml test tube, covered with a cover slip on the tube, and kept standing for 20 min. Then, the cover slip was lifted carefully and placed on the microscope slide for oocyst observation under 40 × and 100 × magnification. A similar method was used for stool and manure samples. Microscopically, *Cryptosporidium* oocysts are spherical or slightly oval in shape, colorless, thick-shelled, and have four elongated sporozoites.

**Fig. 2:**
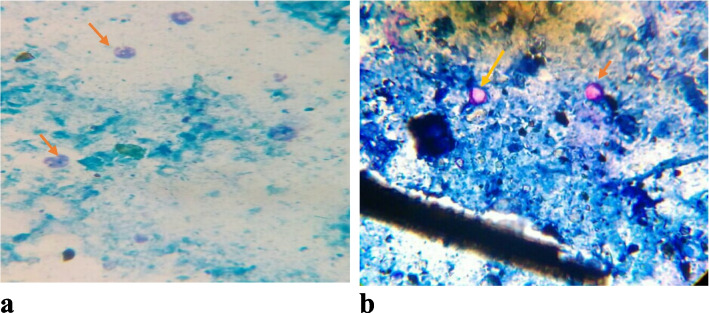
Images of oocysts of *Cryptosporidium*. **a**. *Cryptosporidium* oocyst (oval, red) from cattle feces in modified Ziehl–Neelsen staining method (100X): **b***.Cryptosporidium* oocysts (round, pinkish) from sheep feces in Modified Ziehl–Neelsen staining method (100x)

Additionally, thin smear was prepared from fecal, stool, and manure samples following the procedure described by Casemore [36]. For manure smear preparation, the dry sample (manure) were mixed well using a drop of water to create a uniform suspension. Once the sample was well mixed, it was distributed on a microscope slide using plastic stick. Two thin smears were prepared for each of manure samples to increase the detection of *Cryptosporidium* oocysts. The prepared smear was air dried and fixed with concentrated methanol for three minutes. After staining by carbolfuschin for twenty minutes, the smears were washed in running water for 1–2 min. It was then decolorized for 30 s in 1% hydrsochloric acid in ethanol and counterstained for one minute in 3% methylene blue. Finally, the slides were examined under a microscope at 100 × objective lenses. The oocysts were identified according to standard methods, which appear as pink-stained, round to oval structures of about 3 to 6 µm in diameter, containing distinct internal structures (Fig. 2). The intensity of *Cryptosporidium* oocysts was determined semi-quantitatively in modified Ziehl–Neelsen stained smear by counting the average number of oocysts in 10 randomly selected fields (oocysts per field) at 100 × magnification. The intensity of oocyst was graded as low (1–5 oocyst), medium (6–10 oocyst), and high (above 10 oocysts) [37].

### Data management and analysis

All data collected from study sites and laboratory results were entered and stored in a Microsoft Excel spreadsheet program. R statistical software (Version 4.2.0) was used to analyze data of dairy cattle, sheep, manure, and humans separately. For all study subjects, the associations of explanatory variables with the likelihood of *Cryptosporidium* infection were determined first by univariable logistic regression. For cattle data, variables having p ≤ 0.25 were selected for further multivariable logistic regression to identify the potential risk factors using a stepwise selection procedure [38]. A variance inflation factor was calculated for each independent variable of study subjects, and values less than 10 were chosen for model construction in multivariable logistic regression. Data analysis for sheep, human and manure was based univariable logistic analysis as only one variable for each was found significant results were considered statistically significant when the p ≤ 0.05 at a 95% confidence interval.

## Results

### Prevalence of Cryptosporidium infection

All collected samples were examined microscopically. The prevalence of *Cryptosporidium* in dairy cattle, sheep, humans, and manure was 20.5% (95% CI: 15.5, 26.3), 14% (95% CI: 9.5, 19.6), 16% (95% CI: 7.6, 28.3), and 13.2% (95% CI: 6.2, 23.6), respectively (Table 1).

**Table 1:** Overall prevalence of *Cryptosporidium* infection

Study subject	Study site	Number examined	Number of positive	Percentage (%)	95% Confidence interval
Dairy cattle	Adama	132	25	18.9	12.6, 26.7
	Asella	102	23	22.5	14.9, 31.9
	Total	234	48	20.5	15.5, 26.3
Sheep	Adama	92	16	17.3	10.3, 26.7
	Asella	108	12	11.1	10.2, 25.1
	Total	200	28	14.0	9.5, 19.6
Human	Adama	25	5	20.0	6.8, 40.7
	Asella	31	4	13.0	3.6, 29.8
	Total	56	9	16.0	7.6, 28.3
Manure	Adama	33	3	9.0	1.9, 24.3
	Asella	35	6	17.1	6.6, 33.6
	Total	68	9	13.2	6.2, 23.6

#### Risk factors for the occurrence of Cryptosporidium infection

Eight independent variables were used to determine the association with the occurrence of *Cryptosporidium* infection in dairy cattle (Table 2). Of these, age, body condition score of animals and farm hygienic conditions showed significant association with *Cryptosporidium* infections in dairy cattle. The occurrence of infection was 3.48 times higher in calves aged 1–12 months (OR = 3.48, 95%CI = 1.61–8.05) than in those aged above 36 months. Infection was 3.07 times higher in the age group of 13–36 months (OR = 3.07, 95%CI = 1.22–7.93) than those above 36 months. Similarly, the occurrence of *Cryptosporidium* infection in cattle with poor body condition was 4.51 times higher than animals with a good body condition score. Furthermore, cattle from farms having poor hygienic conditions were 3.67 times more likely to be infected than cattle in good hygienic conditions. Other factors (sex, breed, management system, agro-ecology, and fecal consistency) did not show siginificat association with occurrence of *Cryptosporidium* infection.

**Table 2:** Univariate logistic regression of host and management related risk factors for prevalence of *Cryptosporidium* infections in dairy cattle

Variables	Category	Number Examined	Number of positive (%)	Odd ratio	95% Confidence interval	*p*-Value
Age (month)	1–12	91	26 (28.6)	3.48	1.61 – 8.05	0.002*
	13–36	46	12 (26)	3.07	1.22 – 7.93	0.018*
	> 36	97	10 (10.3)	Ref		
Sex	Female	182	40 (22.0)	0. 65	0.26 – 1.4 2	0.302
	Male	52	8 (15.4)	Ref		
Body condition	Poor	9	4 (14.4)	4.51	1.01–19.2	0.039
	Medium	132	30 (22.7)	1.66	0.84–3.42	0.156
	Good	93	14 (15)	Ref		
Breed	Pure	154	29 (18.8)	1.06	0.44 – 2.83	0.899
	Local	41	12 (29)	1.89	0.67 – 5.70	0.238
	Cross	39	7 (17.9)	Ref		
Farm hygiene	Poor	20	9 (45)	3.67	1.39 – 9.48	0.007*
	Good	214	39 (18.2)	Ref		
Agro-ecology	Midland	132	25 (18.9)	0.80	0.42 – 1.52	0.498
	Highland	102	23 (22.5)	Ref		
Management system	Semi-intensive	82	14 (17.1)	0.71	0.35 – 1.40	0.340
	Intensive	152	34 (22.4)	Ref		
Fecal consistency	Normal	146	30 (20.5)	2.07	0.36 – 39.14	0.501
	Soft	79	17 (21.5)	2.19	0.36 – 42.14	0.473
	Diarrhea	9	1 (1.1)	Ref		
Total observation		234	48(20.5)			

Key: *Represent statistically significant difference (*p* ≤ 0.05)

Table 3 shows factors associated with *Cryptosporidium* infection in sheep. From five independent variables/predictors included in univariate logistic regression, age has been significantly associated with the likelihood of *Cryptosporidium* infection. Accordingly, sheep aged 1–6 months (OR = 2.69, 95%CI = 1.19–6.38, *p* = 0.020) were 2.69 times more likely to be exposed to *Cryptosporidium* infection than those aged above 6 months. In this study, other variables such as sex, body condition, agro-ecology, and fecal consistency were not significantly associated with the occurrence of infection.

**Table 3:** Univariate logistic regression of host and environment related risk factors for prevalence of Cryptosporidium infections in sheep

Variables	Category	Number Examined	Number of positive (%)	Odd ratio	95% Confidence interval	*p*-Value
Age (months)	1- 6	87	18 (20.7)	2.69	1.19 – 6.38	0.020*
	> 6	113	10 (8.8)	Ref		
Sex	Male	94	11 (11.7)	0.69	0.30 – 1.55	0.380
	Female	106	17 (16.0)	Ref		
Body condition	Medium	115	15 (13.0)	1.07	0.46 – 2.55	0.871
	Poor	2	2 (100)	2.56	0.50 – 10.47	0.211
	Good	83	11 (13.3)	Ref		
Agro-ecology	Midland	92	16 (17.4)	1.68	0.76 – 3.85	0.205
	High land	108	12 (11.1)	Ref		
Fecal consistency	Normal	181	22 (12.2)	0.14	0.01 – 3.58	0.167
	Soft	17	5 (29.4)	0.42	0.01 – 11.94	0.562
	Diarrhea	2	1 (50.0)	Ref		
Total observation		200	28(14)			

Key: *Represent statistically significant difference (*p* ≤ 0.05)

In univariable logistic regression, manure type was significantly associated with the occurrence of *Cryptosporidium* oocyst (Table 4). The occurrence of *Cryptosporidium* oocyst was 22.80 times higher in cattle manure (OR = 22.80, 95%CI = 3.60–198.92, *p* = 0.001) than manure of sheep.

**Table 4:** Univariate logistic regression for occurrence of *Cryptosporidium* oocysts in manure

Variables	Category	Number examined	Number of positive (%)	Odd ratio	95% Confidence interval	*p*-Value
Manure types	Cattle manure	6	4 (66. 7)	22.80	3.60 – 198.92	0.001*
	Sheep manure	62	5 (8.1)	Ref		
Agro-ecology	Highland	35	6 (17.1)	2.07	0.50 – 10.54	0.335
	Midland	33	3 (9.1)	Ref		
Total observation		68	9 (13.2)			

Key: *Represent statistically significant difference (*p* ≤ 0.05)

Only one variable, occupation (*p* = 0.046) was significantly associated with the occurrence of *Cryptosporidium* infection in human (Table 5). The likelihood of *Cryptosporidium* infection in individuals working on dairy farms (OR = 13.14, 95%CI = 1.12–306.22, *p* = 0.046) was 13.14 times higher when compared to smallholder farmers.

**Table 5:** Univariate logistic regression of human-related risk factors for prevalence of *Cryptosporidium* infections

Variables	Category	Number of Examined	Number of positive (%)	Odd ratio	95% CI	*p*-value
Age	Young	21	2(9)	0.42	0.06 – 1.97	0.312
	Adult	35	7(20)	Ref		
Occupation	Farm worker	3	2(67)	13.14	1.12 – 306.22	0.046*
	Smallholders	53	7(13)	Ref		
Sex	Male	46	8(17.4)	1.89	0.29 – 37.45	0.569
	Female	10	1(10)	Ref		
Total observation		56	9(16)			

Key: *Represent statistically significant difference (*p* ≤ 0.05)

Multivariable logistic regression analysis was used to identify the potential risk factors for the occurrence of *Cryptosporidium* infection in dairy cattle, sheep, humans, and manure samples (Table 6). For dairy cattle, age, farm hygiene, and body condition of animals were entered into multivariate regression. In sheep, only the age of the animal was indicated as a potential risk factor for *Cryptosporidium* infection in multivariate regression. Occupation and manure types were significantly associated with the occurrence of *Cryptosporidium* infection in humans and manure samples, respectively.

**Table 6:** Multivariable logistic regression model of potential risk factors for the occurrence of *Cryptosporidium* in dairy cattle

Variables	Category	Odd Ratio	95% Confidence interval	*p*-Value
Farm hygiene	Poor	3.67	1.39 – 9.5	0.007*
	Good	Ref		
Age (month)	1–12 months	2.60	1.13 – 6.27	0.027*
	13–36 months	2.93	1.15 – 7.61	0.024
	> 36 months	Ref		

Key: *Represent statistically significant difference (p ≤ 0.05)

#### Intensity of Cryptosporidium oocyst

**Fig. 3:**
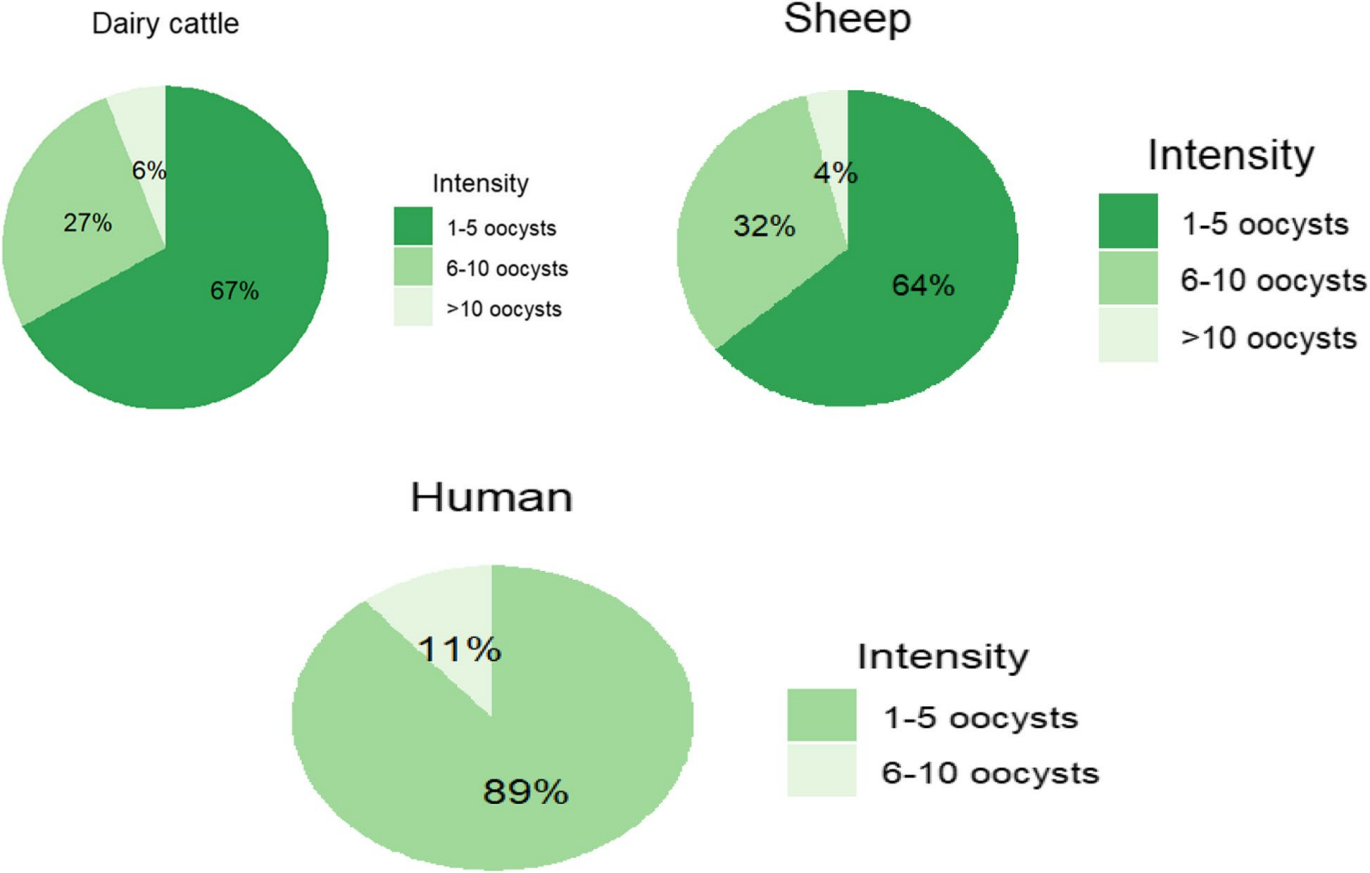
Pie charts shows the intensity of *Cryptosporidium* oocysts. The highest intensity of *Cryptosporidium* oocysts (> 10 oocysts) per field were recorded in dairy cattle (3/48) and sheep (1/32). The low intensity level were recorded in human samples (8/9)

For all positive results, the average oocyst count per field was determined by selecting 10 fields of 100 × magnification randomly (Fig. 3). In the dairy cattle sample, 32 showed an average of 1–5 oocysts, 13 showed an average of [6–10] oocysts, and 3 showed an average of > 10 oocysts) from 48 positive results. Similarly, 28 positive samples were examined for intensity in sheep, showing an average of (1–5 oocysts) in 18 samples, 9 (6–10 oocysts), and 1 (> 10 oocysts) per field. Furthermore, nine positive samples were examined for intensity in human samples and the average of an oocyst recorded was eight (1–5 oocysts), and one (6–10 oocysts). All of the nine manure samples showed an average of 1–5 oocysts. The highest average oocyst count was recorded in samples from dairy cattle.

#### Age related intensity of Cryptosporidium oocyst in dairy cattle and sheep

**Fig. 4:**
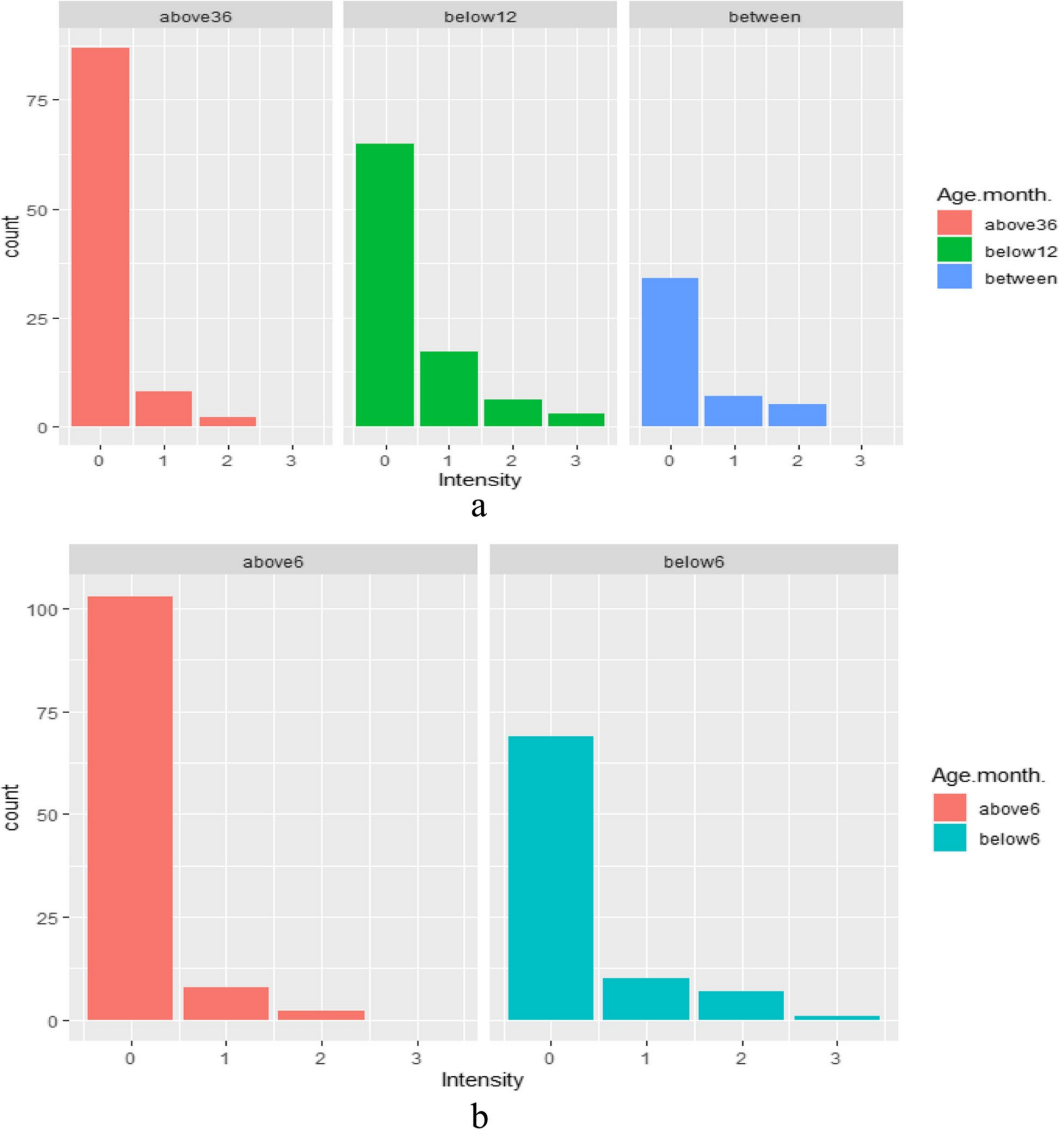
Bar graph shows an age related intensity of *Cryptosporidium* oocyst in dairy cattle and sheep. Intensity: 1 = (1–5 oocysts), 2 = (5–10 oocysts), 3 = (> 10 oocysts). **a**. A higher average level of oocyst intensity (> 10 oocysts) per-fields showed in dairy calves age of 1–12 months. **b**. similarly, the highest oocyst intensity recorded in sheep lambs (1–6 months)

A higher average level of oocyst intensity (> 10 oocysts) per field was examined in dairy cattle aged 1–12 months (Fig. 4). The moderate (6–10 oocysts) and lower average of oocyst counts (1–5 oocysts) per field were examined at ages of 12–36 months and above 36 months, respectively. Similarly, higher average oocyst counts (> 10 oocysts) per field were examined in sheep at an age of 1–6 months, followed by moderate intensity (6–10 oocysts) at an age of over six months.

#### Manure handling practices in dairy farm owners' and farmer's households

In manure handling practice, frequency of removal, place of manure removal, and uses of manure were assessed during sample collection from dairy farms and small holder farmers (Table 7). Only 16.1% of respondents dispose the manure daily from their animal houses. 5% of them had storage holes, and 8.8% dispose the manure on grazing land. Nearly all of them (98.5%) used manure for crop production, and all of them did not apply any manure treatment.

**Table 7:** Manure handling practices in dairy farm owners' and farmer's households

Variables	Category	Number of respondents	Percentage (%)
Frequency of manure disposal	Monthly	21	30.8
	Weekly	36	52.9
	Daily	11	16.1
Place of manure disposal	Grazing land	6	8.8
	Manure storage hole	4	5.8
	Crop land	57	85.3
	Biogas tank	1	1.4
Use of manure	Crop production	67	98.5
	Energy production	1	1.4
Application of manure treatment	Yes	0	0
	No	68	100

## Discussion

The overall prevalence of *Cryptosporidium* infection in dairy cattle was 20.5%, which is comparable to other studies from Ghana [39], Nigeria [40], and Iraq [41]. However, the current result is higher than the report of Wegayehu et al. [27], who reported from cattle reared under an extensive management system. Geurden et al.[42], also reported a higher prevalence in intensive-managed cattle than in extensively managed cattle. This suggests that cryptosporidiosis is more prevalent in dairy farms under intensive management systems compared to the extensively reared cattle. Low prevalence in cattle under extensive management systems can be due to their lower exposure to infection where oocysts are dispersed on a large surface and are exposed to direct sunlight, which reduces the oocysts' viability, resulting in a reduced infection pressure [43].

Observed higher prevalence of infection in young (1–12 months) age group is in line with the report from Haramaya [22] and Southeastern Ethiopia [44], Nigeria [45], and Brazil [46]. However, lower seroprevalence figures have been also reported from different parts of Ethiopia: Tigrai, Bishoftu and West Showa [23, 47, 48]. The prevalence in the (13–36 months) age groups is comparable with the findings from Ghana [39] and Iraq [41]. Association of age with diseases occurrence have been also reported by Ayana et al.[23]; Ebiyo and Haile [49]; Venu et al. [50]; and Santín et al. [51], which stated the importance of age in the occurrence of *Cryptosporidium* infection. This is explained by the underdeveloped immune system of calves that decrease their resistant to infection by *Cryptosporidium* infection [20]. The current study also confirms that adult dairy cattle (above 36 months) shed *Cryptosporidium* oocyst indicating their role in environmental contamination. Similarly, Díaz et al. [20] explain that adult cattle have a role in the outbreak of cryptosporidiosis in calves and humans, even though healthy calves under one month are the main carriers of *Cryptosporidium* species. Other authors also found that the level of oocyst shedding peaks during parturition as a result of immune depression and increases the risk of infection for their new born [52], highlighting the importance of adult cattle as reservoir and their contribution to the transmission and environmental contamination[10]. Farm hygiene is another risk factor that showed an association with the occurrence of *Cryptosporidium* infection in dairy cattle. Locally, Ayele et al. [53], Abebe et al. [54], and Adinew and Geremew [49] reported similar findings from different parts of the country. Similarly, Castro-hermida et al. [55] indicated that the risk of exposure to Cryptosporidium infection decreases with cleaning frequency of their house. Poor hygienic conditions create a dirty and muddy environment, which is favorable for the survival of oocysts and environmental contamination with subsequent exposure of susceptible animals.

On the other hand, the prevalence of *Cryptosporidium* infection in sheep was comparable with the reports from Iran [56] and Poland [57] and Italy [37]. Higher prevalence figures have been also reported from Mexico [58], Brazil [59] and Iraq [60]. These discrepancies in prevalence might be attributed to the management system in which the present study collected the sample from extensively reared sheep. The sampling animals (sheep) in this study are kept out of doors and are less exposed to *Cryptosporidium* oocyst than intensively managed sheep, whose crowding and confinement to small areas favor environmental contamination with oocyst and exposure to infection [59]. Furthermore, in an extensive management system, the oocyst spreads over a large surface area, resulting in low density and infection pressure [42].

The occurrence of infection is more likely high in sheep under 6 months than above 6 months similar to findings from other countries, including Spain [61], Nigeria [62], and England [63]. The current finding is comparable with the findings of Regassa et al.[22] and Dinka and Berhanu [47] from Ethiopia. It is also found to be higher than the reports by Wegayehu et al. [24].The variation might be due to the difference in methodological method; the present study used microscopical examination versus the highly sensitive molecular method used by the former authors. The prevalence of infection in sheep of age above 6 months was in line with Venu et al. [48], from sheep lambs. However, it is lower than the findings of Castro-Hermida et al. [61] and Abare et al.[62]. This finding indicates that adult sheep may serve as a reservoir for *Cryptosporidium* infection and increase the risk of infection for their lambs. Similarly, Firoozi et al. [64] and Dessì et al. [37] support the finding that there is a high risk of maternal transmission in lambs that live with their dams. Furthermore, Chikweto et al. [65] explained the role of adult sheep as a source of environmental contamination by producing a large volume of feces.

In humans, *Cryptosporidium* oocyst was detected in nine of them which is comparable with the reports of Tekle.Y [66]. A higher prevalence was reported from individual contact with animals in Ethiopia [25]. Similarly, in this study the occurrence of *Cryptosporidium* infection was greater in dairy farm workers than in smallholder farmers rearing sheep in their houses. Siwila et al. [67] also agree with the current findings; dairy farm workers were more likely to be infected with *Cryptosporidium* than their household members not working on the farm. This is due to the fact that dairy farm workers have frequent contact with their animals, and mostly involved in manure removal or contact which increases the risk of infection from zoonotic *Cryptosporidium* species. Even if the current study has not been done on species identification in both humans and animals, different studies using molecular methods have found a greater prevalence of *Cryptosporidium* infection in people who have had contact with animals [68].

Furthermore, the prevalence of *Cryptosporidium* oocysts in manure in the present study suggests its significance in further environmental contamination and being role as source of infections for animals and humans. Relatively higher prevalence figures have been reported by Lasprilla-Mantilla et al. [17]. Parasite in manure prevalence and oocyst load might vary depending on handling practices, exposure to sun light and moisture content, animal species. A significant association of manure of animal species with the occurrence of *Cryptosporidium* in cattle manure than that of sheep. Pam et al. [13] also reported higher percentage of *Cryptosporidium* oocyst in cattle manure than in sheep manure. Similarly, Fleming et al. [69] found that manure from dairy farms had a higher prevalence of *Cryptosporidium* oocyst than from swine farms. The global *Cryptosporidium* oocyst load indicates that cattle are the most abundant source of oocyst for the environmental contamination, followed by sheep, goats, swine, and other domestic animals [70].

Assessment of manure handling practices among dairy farmers and smallholders showed improper dispose and use animal manures, which leads to environmental contamination. Even though the role of livestock manure as a source of environmental contamination was not investigated in Ethiopia, it is the main problem in North America and Europe. The spread of manure on grazing, and cropland promotes the propagation of infection to large areas and drinking water [16]. In addition, the use of untreated animal manure for horticulture or vegetable cultivation increases the risk of contamination by *Cryptosporidium* oocyst and human exposure [71]. This results in indirect transmission of *Cryptosporidium* infection in humans from contaminated feed and water [6]. Cryptosporidium oocysts are resistant to environmental conditions (e.g. light temperature and survive for a months in environments and in animal manures under cool and wet conditions. A study illustrated that contaminated manures from dairy or beef operations were among the major sources of *Cryptosporidium* oocysts for humans and animals, and a major contaminants of crop fields and drinking water sources [18]. Surface transfer from land-applied manures or leaching through the soil to groundwater are two the mechanisms of transfer of the pathogen to drinking or recreational water, in addition to direct fecal deposition. Runoff from polluted field might act as a vehicle for *Cryptosporidium* oocysts to enter water sources. As a result, cattle farms might be a major source of *Cryptosporidium* infection for humans and other animals and require due attention [19].

The highest intensity of oocyst count (above 10 oocysts) was recorded in a fecal sample collected from dairy cattle (6%), followed by a fecal sample from sheep similar to the findings of Vermeulen et al. [70] reported cattle manure to be the most abundant source of oocysts. Age level intensity indicates that young calves (1–12 months) and lambs (1–6 months) showed a higher load of oocyst (above 10) count per field than adult animals. The finding was supported by Brook et al. [72] who found moderate to intense oocyst shedding in dairy calves. Findings from naturally infected calves also indicate the number of oocysts shed decreases as the age of the calf [73], suggesting susceptibility to infection and oocyst shedding decreases with the age of the animals. A moderate oocyst count (2–6 oocysts) was also recorded in fecal samples from adult cattle and sheep. According to Scott et al. [14] and Pam et al. [13] asymptomatic weaned and adult cattle shed millions of oocysts into the environment. Report from another study also showed that adult ewes shed about 1 × 10^6^ *Cryptosporidium* oocysts into the environment, which peaks during the lambing season [74]. The large volume and fibrinous nature of adult feces, however, reduce the concentration of oocyst and limit the threshold level examined under the microscope [10]. This underestimates the prevalence and oocyst load of *Cryptosporidium* in adult animals and their contribution to the transmission of infection to young animals. Thus, it is important to emphases the role of adult animals in environmental contamination and subsequent transmission of infection, and this point to the need to improve diagnostic techniques used to detect the minimum level of oocyst concentration [75].

## Conclusion

In conclusion, the current study revealed the occurrence of *Cryptosporidium* among in animal-humans-environment interface. The infection was higher in young animals (calves and lambs) compared to adults. Adult animals had also considerable percentage of *Cryptosporidium* infection, which implies their roles in shedding oocysts into the environment and act as a source of infection for their young’s as well as human beings. Age of animals and farm hygiene were identified as major risk factors for the occurrence of *Cryptosporidium* in animals. Young calves and lambs had a high risk of infection due to poorly developed immunity systems and thus required more attention from dairy farmers and sheep owners to prevent mortality and low growth rate. The study also revealed a higher prevalence of *Cryptosporidium* infection in human subjects who have frequent contact with animals and their feces. More importantly, detection of *Cryptosporidium* oocyst in manure samples, indicates the importance of *Cryptosporidium* in environmental contamination and subsequent public health risks. Furthermore, dairy farmers and smallholder farmers dispose of animal manure on agricultural and grazing lands without any treatment, which contaminate water sources. Additionally direct application of untreated manure to agricultural lands and vegetable plots is also a common practices in the area, and certainly contaminate agricultural produce such as vegetables which are often consumed raw or undercooked. Thus, *Cryptosporidium* infection is being regarded as emerging zoonotic and foodborne diseases and adopting one-health approach for its control and prevention is vitally important. Increasing awareness on manure management and proper handling among dairy farms and smallholder farmers are vitally important in minimizing the disease risk.

## Limitation of the study

The study attempted to show the occurrence of *Cryptosporidium* in animal-humans-environment interface. However, it was based on limited number of human subjects and manure samples in addition to use of purposive sampling for various study units. For future studies, molecular characterization and *Cryptosporidium* species identification helps to determine the major species of animal and human health importance in the area.

## Data Availability

The datasets generated and analyzed during the current study are not publicly available due confidentiality of study subjects but are available from the corresponding author on reasonable request.
